# Clinical Implications of Species Identification in Monomicrobial *Aeromonas* Bacteremia

**DOI:** 10.1371/journal.pone.0117821

**Published:** 2015-02-13

**Authors:** Chi-Jung Wu, Po-Lin Chen, Po-Ren Hsueh, Ming-Chung Chang, Pei-Jane Tsai, Hsin-I Shih, Hsuan-Chen Wang, Pei-Hsin Chou, Wen-Chien Ko

**Affiliations:** 1 National Institute of Infectious Diseases and Vaccinology, National Health Research Institutes, Tainan, Taiwan; 2 Institute of Clinical Medicine, National Cheng Kung University College of Medicine, Tainan, Taiwan; 3 Departments of Internal Medicine, National Cheng Kung University Hospital, Tainan, Taiwan; 4 Emergency Medicine, National Cheng Kung University Hospital, Tainan, Taiwan; 5 Departments of Laboratory Medicine, National Taiwan University Hospital and College of Medicine, Taipei, Taiwan; 6 Internal Medicine, National Taiwan University Hospital and College of Medicine, Taipei, Taiwan; 7 Departments of Medicine, National Cheng Kung University College of Medicine, Tainan, Taiwan; 8 Biochemistry and Molecular Biology Medicine, National Cheng Kung University College of Medicine, Tainan, Taiwan; 9 Medical Laboratory Science and Biotechnology, National Cheng Kung University College of Medicine, Tainan, Taiwan; The University of Hong Kong, HONG KONG

## Abstract

**Background:**

Advances in *Aeromonas* taxonomy have led to the reclassification of aeromonads. Hereon, we aimed to re-evaluate the characteristics of *Aeromonas* bacteremia, including those of a novel species, *Aeromonas dhakensis*.

**Methodology/Principal Findings:**

A retrospective study of monomicrobial *Aeromonas* bacteremia at a medical center in southern Taiwan from 2004–2011 was conducted. Species identification was based on *rpoB* sequencing. Of bacteremia of 153 eligible patients, *A. veronii* (50 isolates, 32.7%), *A. dhakensis* (48, 31.4%), *A. caviae* (43, 28.1%), and *A. hydrophila* (10, 6.5%) were the principal causative species. *A. dhakensis* and *A. veronii* bacteremia were mainly community-acquired and presented as primary bacteremia, spontaneous bacterial peritonitis, or skin and soft-tissue infection, whereas *A. caviae* was associated with hospital-onset bacteremia. The distribution of the AmpC β-lactamase and metallo-β-lactamase genes was species-specific: *bla*_AQU-1_, *bla*_MOX_, or *bla*_CepH_ was present in *A. dhakensis*, *A. caviae*, or *A. hydrophila*, respectively, and *bla*_CphA_ was present in *A. veronii*, *A. dhakensis*, and *A. hydrophila*. The cefotaxime resistance rates of the *A. caviae*, *A. dhakensis*, and *A. hydrophila* isolates were higher than that of *A. veronii* (39.5%%, 25.0%, and 30% *vs.* 2%, respectively). *A. dhakensis* bacteremia was linked to the highest 14-day sepsis-related mortality rate, followed by *A. hydrophila*, *A. veronii*, and *A. caviae* bacteremia (25.5%, 22.2%, 14.0%, and 4.7%, respectively; *P* = 0.048). Multivariate analysis revealed that *A. dhakensis* bacteremia, active malignancies, and a Pitt bacteremia score ≥ 4 was an independent mortality risk factor.

**Conclusions/Significance:**

Characteristics of *Aeromonas* bacteremia vary between species. *A. dhakensis* prevalence and its associated poor outcomes suggest it an important human pathogen.

## Introduction

*Aeromonas* species are aquatic gram-negative bacilli that are ubiquitously distributed in natural environments and implicated in a variety of human diseases [1]. Previous studies have indicated that three *Aeromonas* species, *Aeromonas hydrophila*, *A*. *caviae*, and *A*. *veronii* bv. sobria, accounted for > 95% of all *Aeromonas* blood-borne infections, and liver cirrhosis and malignancies are two well-known predisposing conditions associated with *Aeromonas* bacteremia [1,2]. Continuing advances in the field of *Aeromonas* taxonomy have led to the reclassification of aeromonads. Recent phylogenetic analyses indicated that *A*. *aquariorum*, a species first described in 2008, and *A*. *hydrophila* subsp. *dhakensis* are both incapable of fermenting arabinose and belong to the same taxon [3,4]. Therefore, a formal reclassification of both species as *A*. *dhakensis* sp. nov. comb nov. was proposed [3]. Although human *A*. *dhakensis* infections have been reported, their clinically relevant characteristics have not been thoroughly established [5–8]. *A*. *dhakensis* can be clearly differentiated from *A*. *hydrophila* by its *gyrB*, *rpoB*, or *rpoD* gene sequences, its inability to ferment arabinose, and the cluster analysis of spectra generated by matrix-assisted laser desorption ionization-time of flight mass spectrometry (MALDI-TOF) [6,7,9,10]. Our earlier work also showed that *A*. *dhakensis* exhibits higher pathogenicity than *A*. *hydrophila*, which justifies the need to further differentiate *A*. *dhakensis* from *A*. *hydrophila* [11,12].

To date, the clinically relevant characteristics of *A*. *dhakensis* bacteremia have not been well described. Due to the changing taxonomy, we re-evaluated these characteristics and antimicrobial resistance profiles of causative isolates associated with monomicrobial *Aeromonas* bacteremia, with consideration of the novel species *A*. *dhakensis*.

## Methods

### Patients and definition

A retrospective study of adults (age ≥ 18 years) with monomicrobial *Aeromona*s bacteremia at the National Cheng Kung University Hospital, a medical center in southern Taiwan, was conducted between 2004 and 2011 and was approved by the Institutional Review Board (B-ER-101-031) of the study hospital. Clinical information was retrieved from medical records. The patient information were anonymized and de-identified prior to analysis, and therefore the requirement for informed consent was waived by Institution Review Board.

Monomicrobial *Aeromonas* bacteremia was defined as the presence of an *Aeromonas* species in at least one blood culture from a patient with symptoms or signs of infection. Patients in which different species or multiple *Aeromonas* species were isolated from the blood were excluded. If a patient experienced more than one episode of *Aeromonas* bacteremia due to genetically related *Aeromonas* strains, all instances were counted as one episode. Community-onset infections were defined as those with the first positive blood culture collected within 48 hours after admission; the remaining infections were defined as hospital-onset infections. Those without apparent infection sites were defined as primary bacteremia. The severity of any underlying medical illness was determined as fatal, ultimately fatal, or nonfatal, according to the McCabe score [13]. The severity of the bacteremia on the day of onset was graded by the *Pittsburgh (Pitt) bacteremia* score, and critical illness was defined as a score of at least 4 points [14]. Steroid use was defined as the receipt of corticosteroid treatment (10 mg or an equivalent daily dosage) for more than 2 weeks. Recent antineoplastic chemotherapy or antimicrobial therapy was defined as the receipt of cancer chemotherapy or an oral or parenteral antimicrobial agent for > 48 hours within 2 weeks of the onset of bacteremia. Antimicrobial regimens given before the susceptibility results became available were defined as empirical therapy, whereas those subsequently adjusted accordingly were defined as definitive therapy. Appropriate drugs were those with demonstrable *in vitro* activity against the causative isolates. Breakthrough bacteremia was defined as a bacteremic episode occurring at least 48 hours after initiating antimicrobial therapy. Sepsis-related mortality was the death of a patient with a clinical course suggestive of persistently active infection without obvious other explanations for death.

### *Aeromonas* species identification

*Aeromonas* blood isolates were stored at -70°C until use. The *Aeromonas* isolates were identified by a positive oxidase test, D-glucose fermentation, motility, the absence of growth in 6.5% sodium chloride, and resistance to the vibriostatic agent O/129 (150 μg), and by using the Vitek GNI Plus system (BioMérieux Marcy-l’Etoile, France). Species identification of each *Aeromonas* isolates was determined based on sequence analysis of partial *rpoB* with the PCR primers Pasrpob-L and Rpob-R and additional *rpoD* for *A*. *dhakensis* with the primers 70F and 70R [15,16]. The amplified sequences were compared with reference sequences from the GenBank database using a BLAST (http://www.ncbi.nlm.nih.gov/BLAST/) search of homologous sequences. Based on sequence analyses, the isolates with a dissimilarity value ≤ 2.0% for a given strain were identified as that strain [1]. L-arabinose fermentation was utilized in the species differentiation between *A*. *dhakensis* and *A*. *hydrophila* [3,7]. Genetic relatedness of isolates was examined by arbitrarily primed PCR with the primers ERIC-1R and ERIC-2R [17].

### Antimicrobial drug susceptibility testing

The minimum inhibitory concentrations (MICs) of antimicrobial agents for *Aeromonas* isolates were determined by the broth microdilution method using the Trek Sensititre system (Trek Diagnostics, West Essex, England), and the results were interpreted following the Clinical and Laboratory Standards Institute (CLSI) recommendations for *A*. *hydrophila* complex [18]. The criteria for doxycycline and piperacillin susceptibility followed the CLSI recommendation for *Enterobacteriaceae* [19]. In isolates not susceptible to cefotaxime, ceftriaxone, or ceftazidime, the extended-spectrum β-lactamase (ESBL)-producing phenotypes were examined as previously described [20]. Resistance to broad-spectrum cephalosporins was defined as resistance to at least one third-generation cephalosporin, *i*.*e*., cefotaxime, ceftriaxone, or ceftazidime.

### Detection of the AmpC and metallo-β-lactamase (MBL) genes

The study isolates were screened for the genes encoding AmpC β-lactamases (AQU-1, MOX-like, and CepH β-lactamases) and CphA (MBL) by a colony hybridization assay with digoxigenin-labeled probes (DIG DNA Labeling kit and Nucleic Acid Detection Kit, Roche, Germany). The probe for detection of *bla*_AQU-1_ consisted of *bla*_AQU-1_ amplified from *A*. *dhakensis* AAK1with the primers AQU-2F and AQU-2R [21]. The probe for *bla*_MOX_-like genes consisted of a gene sequence 98.0% identical to *A*. *caviae bla*_MOX-6_ (GenBank accession no. GQ152601) amplified from a blood isolate with the primers AcMOX-F and AcMOX-R. The probe for *bla*_CepH_-like genes consisted of a gene sequence 95.5% identical to *A*. *hydrophila bla*_CepH_ (GenBank accession no. CP000462) amplified from a blood isolate with the primers CepH-F11 and CepH-R1129. The probe for *bla*_CphA_ was previously described [22]. The DNA sequences of the primers used in this study are summarized in Table 1.

**Table 1:** PCR primers used in this study.

Target gene	Primer	Primer sequence (5’–3’)	Reference or sources
rpoB	Pasrpob-L	F (5’-GCAGTGAAAGARTTCTTTGGTTC-3’)	[15]
	Rpob-R	R (5’-GTTGCATGTTNGNACCCAT-3’)	
rpoD	70F	F (5’-ACGACTGACCCGGTACGCATGTAYATGMGNGARATGGGNACNGT-3’)	[16]
	70R	R (5’-ATAGAAATAACCAGACGTAAGTTNGCYTCNACCATYTCYTTYTT-3’)	
AP-PCR^a^	ERIC-1R	F (5’-ATGTAAGCTCCTGGGGATTCAC-3’)	[17]
	ERIC-2R	R (5’-AGTAAGTGACTGGGGTGAGCG-3’)	
aqu-1	AQU-2F	F (5’-GCTATGCTGGCGGCTTTCCAAC-3’)	[21]
	AQU-2R	R (5’-TCAGGG AGCCAGCTTGCTCAG-3’)	
*bla*_MOX_-like gene	AcMOX-F	F (5’-ATGCAACAACGACAATCCATCC-3’)	This study
	AcMOX-R	R (5’-TTACCTGGCCAGTTGCGTCAG-3’)	
*bla*_CepH_-like gene	CepH-F11	F (5’-CCAGAKCCCTGCCACTGCTGGC-3’)	This study
	CepH-R1129	R (5’-AAATGGCATGGGCCGCGCTG-3’)	
cphA	ANY-SSD/F	F (5’-GCTTAGAGCTCCTAAGGAGCAAGATGAAAGGTTGG-3’)	[22]
		R (5’-GCATAGGTACCTTATGACTGGGGTGCGGCCTTG-3’)	

^a^ Arbitrarily primed PCR.

### Statistical analysis

All analyses were performed with the Statistical Package for the Social Sciences version 17.0 (SPSS, Chicago, IL, USA). Continuous variables are expressed as mean values ± standard deviation and were compared by the Analysis of Variance test. Categorical variables were compared by the Fisher exact test or chi-square test. All biologically plausible variables with a *P* value of ≤ 0.20 in the univariate analysis were considered for inclusion in the logistic regression model for multivariate analysis. The time to mortality among patients with bacteremia due to four *Aeromonas* species was analyzed using Kaplan–Meier survival analysis and the log-rank test. A *P* value < 0.05 was considered to be significant, and all tests were 2-tailed.

## Results

There were 160 episodes of monomicrobial *Aeromonas* bacteremia between *2004 and 2011*. Seven cases were excluded due to a lack of available causative isolates. *Seven patients experienced two bacteremic episodes due to distinct Aeromonas isolates confirmed by AP-PCR (repeated bacteremia)*. *Overall*, *153 monomicrobial bacteremic episodes from 146 patients were included*. For convenience, an episode was counted as one case.

Of 153 blood isolates, *A*. *veronii* (50 isolates, 32.7%), *A*. *dhakensis* (48, 31.4%), and *A*. *caviae* (43, 28.1%) were the three principal species involved in bacteremia according to *rpoB* sequencing, followed by *A*. *hydrophila* (10, 6.5%) and *Aeromonas* spp. (2, 1.3%). All 48 *A*. *dhakensis* isolates, also confirmed by *rpoD* sequencing, exhibited the inability to ferment arabinose, whereas all *A*. *hydrophila* isolates were able to ferment arabinose. Some *A*. *veronii*, *A*. *dhakensis*, *A*. *caviae*, and *A*. *hydrophila* isolates were first identified as *A*. *veronii* bv. sobria (41, 82%), *A*. *hydrophila* (46, 95.8%), *A*. *caviae* (41, 95.3%), and *A*. *hydrophila* (10, 100%), respectively, by the Vitek system. Detailed sequence information is described in the supplementary material (S1 Data).

Demographic data and clinical characteristics of the patients are shown in Table 2. Liver cirrhosis (43.8%), especially for *A*. *veronii*, *A*. *dhakensis*, and *A*. *hydrophila* bacteremia, and malignancies (42.5%) were two common underlying diseases. Of *A*. *dhakensis* bacteremia, 70.8% of the episodes were community-acquired infections, and primary bacteremia, spontaneous bacterial peritonitis (SBP), biliary tract infection (BTI), and skin and soft-tissue infection (SSTI) were the major presentations. Of 7 *patients with repeated bacteremia (6 patients with liver cirrhosis and one with leukemia)*, *13 episodes were community-acquired*, *and 7 and 5 of these episodes were caused by A*. *dhakensis and A*. *veronii*, *respectively*. *A*. *caviae* bacteremia was usually associated with hospital-onset infections, especially vascular catheter-related bacteremia, and less critical illness at the onset of bacteremia. Four patients with community-acquired bacteremia recalled contact histories: flame burn injury and frostbite (2 patients, SSTIs), ingestion of contaminated food (1, gastroenteritis) [23] and unboiled water (1, empyema). No clustering of hospital-onset infections was noted.

**Table 2:** Demographic data, underlying conditions, clinical presentations, and treatment outcomes of 153 patients with monomicrobial Aeromonas bacteremia from 2004 to 2011.

Characteristic	Case no. (%)					*P* value
	All n = 153	*A*. *veronii* n = 50	*A*. *dhakensis* n = 48	*A*. *caviae* n = 43	*A*. *hydrophila* n = 10	
Age, mean ± standard deviation (years)	59.8 ± 14.7	62.9 ± 14.3	55.7 ± 16.0	61.3 ± 12.5	57.7 ± 16.3	0.085
Gender, male	91 (59.5)	28 (56)	27 (56.3)	26 (60.5)	8 (80)	0.533
Hospital-onset bacteremia	63 (41.2)	10 (20)	14 (29.2)	33 (76.7)	6 (60)	< 0.001
Co-morbidity						
Liver cirrhosis	67 (43.8)	24 (48)	30 (62.5)	7 (16.3)	5 (50)	< 0.001
Malignancies	65 (42.5)	25 (50)	14 (29.2)	19 (44.2)	7 (70)	0.052
Leukemia/lymphoma/myeloma	17 (11.1)	11 (22)	2 (4.2)	2 (4.7)	2 (20)	0.013
Myelodysplasia/aplastic anemia	6 (3.9)	3 (6)	2 (4.2)	1 (2.3)	0 (0)	0.738
Hepatocellular carcinoma	29 (19.0)	11 (22)	8 (16.7)	6 (14.0)	4 (40)	0.261
Pancreatobiliary cancer	7 (4.6)	1 (2)	0 (0)	6 (14.0)	0 (0)	0.007
Solid cancer, other site	13 (8.5)	2 (4)	4 (8.3)	6 (14.0)	1 (10)	0.401
Diabetes mellitus	36 (23.5)	11 (22)	12 (25.0)	10 (23.3)	2 (20)	0.980
Obstructive biliary disease (stone or stricture)	12 (7.8)	3 (6)	4 (8.3)	3 (7.0)	0 (0)	0.807
Steroid use	9 (5.9)	1 (2)	1 (2.1)	6 (14.0)	1 (10)	0.049
Renal failure on dialysis	5 (3.3)	2 (4)	3 (6.3)	0 (0)	0 (0)	0.364
Rapidly fatal underlying disease (McCabe classification)	9 (5.9)	8 (16)	0 (0)	0 (0)	1 (10)	0.002
Previous procedures or conditions within 2 weeks of bacteremia onset						
Prior antimicrobial therapy	35 (22.9)	8 (16)	16 (33.3)	9 (20.9)	2 (20)	0.221
Endoscopic examination	14 (9.2)	4 (8)	7 (14.6)	2 (4.7)	1 (10)	0.423
Surgery	13 (8.5)	4 (8)	4 (8.3)	5 (11.6)	0 (0)	0.690
Neutropenia	11 (7.2)	8 (16)	1 (2.1)	0 (0)	2 (20)	< 0.001
Port-A catheter	11 (7.2)	2 (4)	1 (2.1)	7 (16.3)	1 (10)	0.046
Indwelling central venous catheter other than Port-A	6 (3.9)	2 (4)	3 (6.3)	1 (2.3)	0 (0)	0.712
Antineoplastic chemotherapy	6 (3.9)	3 (6)	1 (2.1)	2 (4.7)	0 (0)	0.693
Intensive care unit care	4 (2.6)	1 (2)	3 (6.3)	0 (0)	0 (0)	0.268
Sources of bacteremia						
Primary bacteremia, non-neutropenic	89 (58.2)	28 (56)	23 (47.9)	31 (72.1)	6 (60)	0.132
Primary bacteremia, neutropenic	11 (7.2)	8 (16)	1 (2.1)	0 (0)	2 (20)	< 0.001
Biliary tract infection	15 (9.8)	2 (4)	5 (10.4)	7 (16.3)	0 (0)	0.154
Spontaneous bacterial peritonitis	12 (7.8)	3 (6)	8 (16.7)	0 (0)	1 (10)	0.029
Skin and soft tissue infection	10 (6.5)	5 (10)	5 (10.4)	0 (0)	0 (0)	0.122
Vascular-catheter related infection	5 (3.3)	0 (0)	0 (0)	4 (9.3)	1 (10)	0.023
Enteritis	3 (2.0)	2 (4)	2 (4.2)	0 (0)	0 (0)	0.523
Intra-abdominal infection	3 (2.0)	1 (2)	2 (4.2)	1 (2.3)	0 (0)	0.491
Spontaneous bacterial empyema	3 (2.0)	2 (4)	1 (2.1)	0 (0)	0 (0)	0.548
Pneumonia	2 (1.3)	1 (2)	1 (2.1)	0 (0)	0 (0)	0.778
Pitt bacteremia score ≥ 4	31 (20.3)	12 (24)	13 (27.1)	3 (7.0)	3 (30)	0.071
Appropriate empirical antibiotics	123 (80.4)	48 (96)	34 (70.8)	31 (72.1)	9 (90)	0.004
Appropriate definitive antibiotics	121/134 (90.3)	42/45 (93.3)	35/38 (92.1)	35/41 (85.4)	8/8 (100)	0.439
Mortality rate						
14-day sepsis-related	23/151 (15.2)	7 (14)	12/47 (25.5)	2 (4.7)	2/9 (22.2)	0.048
Crude in-hospital	36/150 (24.0)	12/49 (24.5)	16/47 (34.0)	6 (14.0)	2/9 (22.2)	0.176

Broad-spectrum cephalosporin resistance rates of *A*. *caviae*, *A*. *dhakensis*, and *A*. *hydrophila* were higher than that of *A*. *veronii* (39.5%, 29.2%, and 30% *vs*. 2%, respectively; *P* ≤ 0.001) (Table 3). A multivariate analysis revealed that preceding β-lactam therapy (≥ 48 hours) within 2 days before bacteremia onset was associated with broad-spectrum cephalosporin resistance (odds ratio [OR] 3.7; 95% confident interval [CI], 1.2–11.2; *P* = 0.022). One imipenem-resistant *A*. *dhakensis* isolate was isolated from a patient during ertapenem treatment for ESBL-producing *E. coli BTI, as described previously [22].* Overall, *A*. *veronii* was susceptible to most drugs, whereas *A*. *caviae* was more likely to be resistant to the drugs tested.

**Table 3:** Distribution of β-lactamase genes detected by colony hybridization and antimicrobial resistance profiles of 153 *Aeromonas* blood isolates, 2004–2011.

	Isolate no. (%)					*P* values
	All isolatesn = 153	*A*. *veronii* n = 50	*A*. *dhakensis* n = 48	*A*. *caviae* n = 43	*A*. *hydrophila* n = 10	
Positive hybridization						
Ambler class C β-lactamase						
*bla*_AQU-1_	48 (31.4)	0 (0)	48 (100)	0 (0)	0 (0)	< 0.001
*bla*_MOX_	45 (29.4)	0 (0)	0 (0)	43 (100)	2 (20)	< 0.001
*bla*_CepH_	12 (7.8)	0 (0)	0 (0)	2 (4.7)	10 (100)	< 0.001
Ambler class B β-lactamase						
*bla*_CphA_	109 (71.2)	50 (100)	48 (100)	0 (0)	10 (100)	< 0.001
Antimicrobial resistance						
Cefazolin	149 (97.4)	46 (92)	48 (100)	43 (100)	10 (100)	0.040
Cefuroxime	30 (19.6)	1 (2)	11 (22.9)	15 (34.9)	3 (30)	0.001
Cefoxitin	76 (49.7)	5 (10)	44 (91.7)	25 (58.1)	2 (20)	< 0.001
Cefotaxime	33 (21.6)	1 (2)	12 (25.0)	17 (39.5)	3 (30)	<0.001
Ceftazidime	16 (10.5)	1 (2)	6 (12.5)	7 (16.3)	2 (20)	0.090
Ceftriaxone	35 (22.9)	1 (2)	14 (29.2)	17 (39.5)	3 (30)	< 0.001
Broad-spectrum cephalosporin^a^	35 (22.9)	1 (2)	14 (29.2)	17 (39.5)	3 (30)	< 0.001
Cefepime	1 (0.7)	0 (0)	0 (0)	1 (2.3)	0 (0)	0.470
Aztreonam	4 (2.6)	1 (2)	0 (0)	3 (7.0)	0 (0)	0.188
Ampicillin/sulbactam	151 (98.7)	50 (100)	48 (100)	42 (97.7)	9 (90)	0.060
Piperacillin	20 (13.1)	1 (2)	11 (22.9)	8 (18.6)	0 (0)	0.007
Piperacillin/tazobactam	17 (11.1)	2 (4)	10 (20.8)	5 (11.6)	0 (0)	0.040
Imipenem	2 (1.3)	0 (0)	2 (4.2)	0 (0)	0 (0)	0.226
Meropenem	2 (1.3)	0 (0)	2 (4.2)	0 (0)	0 (0)	0.226
Doxycycline	2 (1.3)	0 (0)	0 (0)	2 (4.7)	0 (0)	0.165
Gentamicin	4 (2.6)	0 (0)	0 (0)	3 (7.0)	1 (10)	0.048
Amikacin	1 (0.7)	0 (0)	0 (0)	1 (2.3)	0 (0)	0.470
Ciprofloxacin	2 (1.3)	0 (0)	0 (0)	2 (4.7)	0 (0)	0.165
Levofloxacin	1 (0.7)	0 (0)	0 (0)	1 (2.3)	0 (0)	0.470
Co-trimethoxazole	24 (15.7)	4 (8)	7 (14.6)	12 (27.9)	0 (0)	0.027
ESBL phenotype^b^	5 (3.3)	0 (0)	0 (0)	4 (9.3)	1 (10)	0.023

^a^ Resistance to at least one third-generation cephalosporin, *i*.*e*., cefotaxime, ceftriaxone, or ceftazidime.

^b^ ESBL = extended-spectrum β-lactamase.

A colony hybridization assay revealed that *bla*_AQU-1_ was constantly present in *A*. *dhakensis* isolates but not in other species, whereas the *bla*_MOX_-like gene was present in all *A*. *caviae* isolates, and the *bla*_CepH_-like gene was present in all *A*. *hydrophila* isolates. The MBL *bla*_CphA_-like gene was present in all *A*. *dhakensis*, *A*. *veronii*, and *A*. *hydrophila* isolates but not in *A*. *caviae* isolates (Table 3).

**Fig 1:**
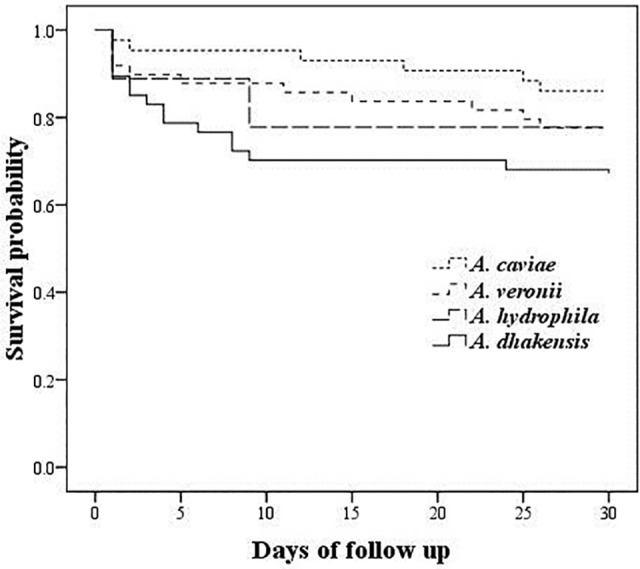
Kaplan-Meier survival curves for 148 patients with monomicrobial bacteremia caused by *Aeromonas veronii*, *A*. *dhakensis*, *A*. *caviae*, and *A*. *hydrophila* (Log-rank test, *P* = 0.02).

The 14-day and in-hospital clinical outcomes were assessed in 151 and 150 patients, respectively. The detailed antimicrobial treatments for these patients are provided in the supplementary material (S1 Data). Patients with *A*. *dhakensis* bacteremia had the highest 14-day sepsis-related mortality rate, followed by bacteremia due to *A*. *hydrophila*, *A*. *veronii*, or *A*. *caviae* (25.5%, 22.2%, 14.0%, and 4.7%, respectively; *P* = 0.048). Inappropriate empirical therapy or definite treatment was not associated with 14-day sepsis-related mortality. A multivariate analysis revealed that a *Pitt bacteremia score* ≥ 4 (OR 44.9; 95% CI 11.0–184.2; *P* < 0.001), *A*. *dhakensis* (OR 8.5; 95% CI 1.9–37.3; *P* = 0.005), and active malignancies (OR 9.1; 95% CI 1.9–43.7; *P* = 0.006) were independent risk factors for 14-day sepsis-related mortality. The Kaplan-Meier survival analysis revealed that *A. dhakensis* bacteremia heralded the worst clinical outcome (log-rank test, *P* = 0.020) (Fig. 1).

Despite receiving appropriate antibiotic treatment, one patient experienced breakthrough bacteremia, and two patients had relapsing bacteremia due to identical strains. Breakthrough *A*. *dhakensis* bacteremia occurred in a patient with a severe *A*. *dhakensis* burn wound infection, and cefotaxime resistance emerged after a 3-day ceftazidime therapy, as described previously [21]. Relapsing bacteremia after a 14-day treatment occurred in a cancer patient with Port A catheter-related *A*. *caviae* bacteremia treated by cefpirome without catheter removal and in a patient with unresectable pancreatic cancer with *A*. *caviae* BTI treated by ciprofloxacin. Overall, of 66 patients infected by cefotaxime-susceptible *A*. *dhakensis*, *A*. *caviae*, or *A*. *hydrophila* strains and treated by a β-lactam for at least 48 hours, only one (1.5%) was later colonized or infected with a resistant strain of the same species.

A subgroup analysis comparing the *A*. *dhakensis* and *A*. *hydrophila* groups revealed that co-morbidity with malignancy and cefoxitin susceptibility were more frequently associated with the *A*. *hydrophila* group (*P = 0*.*027 and P < 0*.*001*, *respectively)*, *and* other clinical *parameters were not significantly different*.

## Discussion

This study revealed that *A*. *dhakensis*, *A*. *veronii* and *A*. *caviae* were the three major species involved in *Aeromonas* bacteremia, while *A*. *hydrophila* played a minor role. Clinical characteristics, antimicrobial resistance profiles, and treatment outcomes of patients with bacteremia varied depending on the species. *A*. *dhakensis* and *A*. *veronii* bacteremia correlate with similar patient characteristics (liver cirrhosis), community acquisition, and infectious diseases (SBP and SSTIs). Manifestations of *A*. *caviae* bacteremia included less involvement of cirrhotic patients and frequent association with hospital-onset infections, as observed in an earlier report [24]. Oral ingestion, replacement of medical devices, or direct contact of abraded wounds with contaminated material can serve as the portals of entry of *Aeromonas* species [1]. We found that patients with either cirrhosis or leukemia experienced repeated episodes of *Aeromonas* bacteremia, which were mostly community-acquired and caused by either *A*. *dhakensis* or *A*. *veronii*. Because of the wide distribution of aeromonads in food products and *A*. *dhakensis* in the environment and the aquatic creatures [1,4,7], food safety and wound hygiene should be emphasized among susceptible hosts to reduce infections. Likewise, microbiological surveillance of hospital water and fluids for medical applications may be considered to prevent *A*. *caviae*-associated hospital-onset infections.

Differences in antimicrobial resistance phenotypes and genotypes are also associated with species variation. The distribution of chromosomal β-lactamases is species-specific among aeromonads, *i*.*e*., class B, C and D in *A*. *hydrophila*, class C and D in *A*. *caviae*, and class B and D in *A*. *veronii* [1,25]. The expression of the three chromosomal β-lactamases is often activated in the presence of inducers or due to the emergence of derepressed mutants [26]. We recently reported that *A*. *dhakensis* intrinsically carries class B, C and D β-lactamases, and AmpC *bla*_AQU-1_ is specific to *A*. *dhakensis* [21,22]. The colony hybridization assay that detected the AmpC and MBL genes in the present study yielded consistent results. Not unexpectedly, broad-spectrum cephalosporin resistance was *associated* with recent *antibiotic* selection pressure, as revealed by the multivariate analysis. Of note, 92% of the *A*. *veronii* isolates were resistant to cefazolin based on the current resistance criterion, *i*.*e*., MIC ≥ 4 μg/ml [18]. Such a result is different from the previous impression of cefazolin susceptibility of *A*. *veronii* [27], which was based on the earlier breakpoint, ≥ 32 μg/ml [28]. Only 2 (1.9%) of *cphA*-carrying *A*. *dhakensis*, *A*. *hydrophila*, and *A*. *veronii* isolates exhibited imipenem resistance, which is in concordance with the finding that CphA carbapenemase production is not easily detected by the conventional *in vitro* susceptibility test, unless using large inocula or a modified Hodge test [22,29].

As in our previous report [30], a low incidence (1.5%) of emergence of broad-spectrum cephalosporin-resistant *Aeromonas* isolates when treating *Aeromonas* bacteremia with a β-lactam. Notably, resistance mainly emerged in the cases of secondary bacteremia due to burn wound infections [21]. Therefore, the use of a broad-spectrum cephalosporin or carbapenem for bacteremia with a high tissue bacterial burden caused by AmpC-carrying or CphA-carrying species should be approached with caution [21,22].

Distinct survival curves were observed among patients with bacteremia due to different *Aeromonas* species. Although individual, phenotypically identified *Aeromonas* species were not shown to predict mortality in earlier reports [2,30], the present study demonstrated that *A*. *dhakensis* bacteremia had the highest mortality rate and was an independent risk factor for mortality, while *A*. *caviae* bacteremia led to the lowest fatality rate. This result is in agreement with the previous finding that *A*. *caviae* exhibits low pathogenic potential, as demonstrated by less toxicity to human cell lines and mice [23,27]. Additionally, higher pathogenicity of *A*. *dhakens*is was demonstrated by increased cytotoxicity to human cell lines and higher lethality to *Caenorhabditis elegans* and mice than *A*. *hydrophila*, *A*. *veronii*, *or A*. *caviae* [5,11,12,23]. Further studies to identify virulence determinants of *A*. *dhakensis* are warranted.

Our results are consistent with previous findings that *A*. *dhakensis* is widely distributed and often misidentified as *A*. *hydrophila* [7,8]. Collectively, the differences in microbiological characteristics, such as the *rpoB*, *rpoD*, or *gyrB* sequencing results, arabinose fermentation ability (negative for *A*. *dhakensis* and positive for *A*. *hydrophila*), MALDI/TOF spectra, and types of the AmpC β-lactamase gene presented herein (*bla*_AQU-1_ for *A*. *dhakensis* and *bla*_CepH_-like gene for *A*. *hydrophila*), support the concept that *A*. *dhakensis* and *A*. *hydrophila* are distinct species [6,7,9,10,21]. Although both *in vitro* and *in vivo* animal models have demonstrated higher pathogenicity of *A*. *dhakensis* than *A*. *hydrophila* [11,12], the comparison of clinical features and treatment outcomes between the cases of *A*. *dhakensis* and *A*. *hydrophila* bacteremia was inconclusive, likely owing to a limited number of the cases of the latter. Clinical studies enrolling more patients are warranted to clarify the issue.

In summary, *A*. *dhakensis*, *A*. *veronii*, and *A*. *caviae* are the three major species that cause *Aeromonas* bacteremia in southern Taiwan. Significant differences existed in their clinical characteristics and antimicrobial resistance profiles. The association with a poor clinical outcome suggests *A*. *dhakensis* an important human pathogen.

## Supporting Information

S1 Data Identification of *Aeromonas* species and antimicrobial therapy. (DOCX)
