# Supplementary material for: Clinical Implications of Species Identification in Monomicrobial Aeromonas Bacteremia
Source: PLoS One. 2015 Feb 13;10(2):e0117821. doi: 10.1371/journal.pone.0117821 (PMC4334500; doi:10.1371/journal.pone.0117821)
Supplement: S1 Data — (DOCX) [file pone.0117821.s001.docx]

**Supplementary Material**

**Data S1**

**Identification of *Aeromonas* species**

According to the *rpoB* sequencing, 50, 48, 43, and 10 isolates were identified as *A. veronii, A. dhakensis, A. caviae*, and *A. hydrophila,* respectively. Of 50 *A. veronii* isolates, 48 and 2 isolates had ≥ 98.8% and 98.0% *rpoB* sequence identity, respectively, with *A. veronii* CIP107763 (GenBank accession no. AY851142.1). All *A. dhakensis* isolates had ≥ 98.4% *rpoB* sequence identity with *A. aquariorum* (prior name *A. dhakensis*) DSM18362T (accession no. FM210471.1). In addition, 45 isolates had ≥ 98.0% *rpoD* sequence identity with *A. aquariorum* DSM18362 (accession no. FJ936132.1), and 3 isolates had ≥ 98.5% *rpoD* sequence identity with *A. hydrophila* subsp. *dhakensis* (prior name of *A. dhakensis*) LMG 19562 (accession no. KC601656.1). All *A. caviae* isolates had ≥ 98.8% *rpoB* sequence identity with *A. punctata* (*caviae*) V83 (accession no. AY851107.1). Of 10 *A. hydrophila* isolates, 8 isolates had ≥ 98.8% *rpoB* sequence identity with *A. hydrophila* ATCC 7966 (accession no. AY851091.1), and 2 isolates had 99.0% *rpoB* sequence identity with *A. hydrophila* F458 (accession no. AY851092.1).

**Antimicrobial therapy**

The 14-day and in-hospital clinical outcomes were assessed in 151 and 150 patients, respectively. With the exclusion of the cases receiving no or inappropriate antibiotics, 122 cases were assessed for therapeutic efficacy of empirical therapy. Of these 122 cases, 67 patients received broad-spectrum cephalosporin-based regimens (monotherapy [63 cases]; in combination with an aminoglycoside [2 cases], a fluoroquinolone [1 case] or doxycycline [1 case]), 30 received 4th generation cephalosporin-based regimens (monotherapy [28 cases] and in combination with doxycycline [2 cases]), and 7 received a carbapenem monotherapy. At 72 hours after bacteremia onset, carbapenem therapy was associated with a higher mortality rate than 3rd or 4th generation cephalosporin-based therapies (42.9% vs. 6.0% or 20%, respectively; *P* = 0.007). However, carbapenem was more often associated with a critical illness, i.e., Pitt bacteremia score ≥ 4 (71.4% vs. 16.2% or 33.3%, respectively; *P* = 0.002).

Most cases (135) were evaluated for therapeutic efficacy of definite therapy, and 116 (86%) cases received an appropriate therapy. The 14-day sepsis-related mortality rates were similar between those with 3rd generation cephalosporin-based regimens (monotherapy [42 cases]; in combination with doxycycline [9 cases] or a fluoroquinolone [2 cases]), 4th generation cephalosporin-based regimens (monotherapy [15 cases]; in combination with doxycycline [1 case]), and fluoroquinolone therapy (monotherapy [35 cases]; in combination with doxycycline [1 case]) (1.9%, 6.3%, and 5.3%, respectively; *P* = 0.592).
